# Membrane-bound IL-12 and IL-23 serve as potent mucosal adjuvants when co-presented on whole inactivated influenza vaccines

**DOI:** 10.1186/1743-422X-11-78

**Published:** 2014-05-03

**Authors:** Tila Khan, Connie L Heffron, Kevin P High, Paul C Roberts

**Affiliations:** 1 Department of Biomedical Sciences and Pathobiology, 1981 Kraft Drive, Corporate Research Center, Virginia Tech, Blacksburg, Virginia 24061, USA; 2 Section on Infectious Diseases, Department of Internal Medicine, Wake Forest University School of Medicine, 100 Medical Center Boulevard, Winston-Salem, NC 27157-1042, USA

**Keywords:** Influenza, Whole inactivated virus vaccine, Adjuvant, Cytokine, IL-12, IL-23

## Abstract

**Background:**

Potent and safe adjuvants are needed to improve the efficacy of parenteral and mucosal vaccines. Cytokines, chemokines and growth factors have all proven to be effective immunomodulatory adjuvants when administered with a variety of antigens. We have previously evaluated the efficacy of membrane-anchored interleukins (IL) such as IL-2 and IL-4 co-presented as *Cyt*okine-bearing *I*nfluenza *Vac*cines (CYT-IVACs) using a mouse model of influenza challenge.

**Findings:**

Here, we describe studies evaluating the parenteral and mucosal adjuvanticity of membrane-bound IL-12 and IL-23 CYT-IVACs in young adult mice. Mucosal immunization using IL-12 and IL-23 bearing whole influenza virus vaccine (WIV) was more effective at eliciting virus-specific nasal IgA and reducing viral lung burden following challenge compared to control WIV vaccinated animals. Both IL-12 and IL-23 bearing WIV elicited the highest anti-viral IgA levels in serum and nasal washes.

**Conclusions:**

This study highlights for the first time the mucosal adjuvant potential of IL-12 and IL-23 CYT-IVAC formulations in eliciting mucosal immune responses and reducing viral lung burden. The co-presentation of immunomodulators in direct context with viral antigen in whole inactivated viral vaccines may provide a means to significantly lower the dose of vaccine required for protection.

## Findings

### Introduction

The use of novel adjuvants, non-replicating virus-like particles (VLP), whole inactivated influenza vaccines (WIV), universal vaccines, and others are potential approaches to address current influenza vaccine challenges [1,2]. Interestingly, in mice WIV have been shown to be more immunogenic and induce superior mucosal IgA responses when administered intranasally (I.N.) compared to split vaccines [3]. Intranasal administration of WIV can lead to B cell dependent hetero-subtypic immunity that is associated with elevated lung and serum sIgA and IgG levels [4]. The use of novel adjuvants in combination with WIV may serve to modulate and enhance both mucosal and systemic immune responses with the potential benefit of antigen sparing and costs associated with vaccine production [5]. Cytokines have great potential to serve as potent and specific adjuvants due to their diverse role in immune responses [6,7]. They are actively being evaluated for use as adjuvants for intranasal vaccine formulations [8] and have been used extensively as soluble adjuvants co-administered with numerous vaccines [9,10], or in a fused or linked form directly with antigen [7,11]. The superior immunogenicity of WIV as compared to split or subunit influenza vaccines [12,13] encouraged us to utilize WIV bearing cytokines as a more immunogenic vaccine formulation.

Previous studies in our laboratory have evaluated the adjuvant potential of membrane-anchored IL-2, IL-4 and GM-CSF directly incorporated on inactivated influenza virus particles in mice and chickens [14,15]. *Cyt*okine bearing *I*nfluenza *Vac*cine (CYT-IVAC) formulations are derived from mammalian cell culture propagation techniques. Here, influenza A virions are harvested from infected MDCK cell lines that are constitutively expressing membrane-bound cytokines at their cell surfaces. As the virus buds and is released they recognize and incorporate the membrane-bound cytokine into the virion envelope. In the present study we expanded our panel of immunomodulators to include IL-12 and IL-23 and evaluated their immunogenicity and protective efficacy by different routes of immunization in young adult mice. IL-12 is a Th1 polarizing cytokine that promotes the differentiation of naïve CD4 + T helper cells towards a Th1 phenotype and stimulates both NK cells and T cells to secrete the immunomodulatory cytokine, IFNγ [16,17]. Co-administration of IL-12 during intranasal vaccination has proven to be efficacious in stimulating enhanced immune responses to pneumococcal [18] and HIV antigens [19] as well as the induction of systemic and mucosal CTL responses [20]. Recently, we demonstrated that both IL-2 and IL-12 can serve as immunopotentiating agents in influenza vaccines tailored for the elderly [21]. Here, we expanded upon those observations and evaluated whether IL-12 and IL-23 in a membrane-bound formulation would serve as mucosal adjuvants for influenza WIV in young adult mice.

## Methods

The cDNA encoding for mouse derived immunomodulators IL-12 and IL-23 was fused inframe to the membrane anchoring regions of the hemagglutinin (HA) of A/WSN/33 influenza virus as previously described [15,21]. The mIL-12 gene derived from pORF-mIL-12 (p35:p40) plasmid (InvivoGen) and the mIL-23 (p19:p40) gene construct synthesized *in vitro* (GenScript®), both encode for a single chain open reading frame in which both subunits are linked by a hydrophobic linker molecule (see Additional file 1: Figure S1). Cytokine gene amplicons were further subcloned into the pcDNA3.1 ~ HA1513 vector using restriction endonuclease sites HindIII and BamHI. Madin-Darby canine kidney cells (MDCK) constitutively expressing membrane-bound IL12/HA and IL23/HA were established and surface expression of membrane bound cytokines was validated by indirect immunofluorescence microscopy as described previously [15,21]. Double gradient purified whole inactivated influenza vaccines (WIV) were produced from influenza A/PR/8/34 (H1N1) virus infected MDCK producer cell lines as described previously and incorporation of membrane-bound cytokines was further validated by western blot analysis of whole viral lysates [15,21]. All animal experiments were performed based on the guidelines of NIH and approval of Institutional Animal Care and Use Committee (IACUC) of Virginia Polytechnic Institute and State University. 8–10 week old female Balb/c mice (*Mus musculus*) from Charles River Laboratories (CRL) were allowed to acclimate for at least 10 days prior to onset of vaccination studies. Lightly anesthetized animals (isoflurane inhalant) were immunized either intramuscularly (I.M.) (0.5 μg/100 μl volume of PBS) or intranasally (I.N.) (3 μg/10 μl volume of PBS into the nostrils) with inactivated double gradient purified control WIV (A/PR/8/34), CYT-IVAC^~mIL12^ and CYT-IVAC^~mIL23^ (n = 5 mice/group). PBS administered intramuscularly (I.M.) served as mock (n = 5 mice/group). On day 21, all mice were administered I.M. a booster dose (I.M. group, 100 ng and I.N. group, 500 ng total viral protein) of vaccine. Blood was collected from the retro-orbital sinus on day 19 and day 35 post-vaccination. Lightly anesthetized animals were challenged intranasally on day 36 post-vaccination with 100 mouse lethal dose 50 (MLD_50:_ 50 μl volume) of mouse-adapted influenza A/PR/8/34 virus. Body weights and clinical symptoms were monitored daily. All animals were sacrificed at day 4 post-challenge to determine viral lung loads. Sera, lungs, and nasal washes were collected post-mortem for biological assays. Quantitative levels of influenza-specific total IgG and IgA antibodies in serum or nasal washes were determined by enzyme-linked immunosorbent assay, ELISA [15]. For standard curve generation, anti-mouse IgG (SouthernBiotech) or anti-mouse IgA (SouthernBiotech) antibodies were serially diluted 2 fold from 160 ng/ml to 0.03125 ng/ml (IgG) or 512 ng/ml to 0.03125 ng/ml (IgA) respectively. Serum samples were diluted 1:20 while nasal washes were undiluted for IgA determination. Quantitation of viral loads in frozen lung samples was determined by tissue culture infectious dose assay [15].

## Results and discussion

Our construct design allows for the expression of murine IL-12(p35p40) and IL-23(p19p40), both heterodimeric cytokines, as single chain full-length IL-12p35p40 and IL-23p19p40 entities joined by a intra-subunit linker segment fused to the 71 amino acid long membrane anchoring region (short stalk, transmembrane and cytoplasmic tail domain) of the HA gene (HA^1513^) derived from influenza A/WSN/33 (H1N1) virus [15] (see Additional file 1: Figure S1). The latter serves to anchor the heterodimeric cytokine within the plasma membrane and is recognized by the virus assembly and budding machinery ensuring packaging within virions. Single chain IL-12 and IL-23 were chosen as they have been shown to be bioactive *in vitro* and *in vivo* using this constellation [22,23]. Following stable transfection of the virus permissive MDCK cell line with recombinant plasmids pcDNA3.1-IL-12(p35p40)/HA^1513^ and pcDNA3.1-IL-23 (p19p40)/HA^1513^) the constitutive cell surface expression of the IL-12 and IL-23 cytokine fusion proteins were confirmed by immunofluorescence microscopy using IL12p40 specific antibodies (see Additional file 1: Figure S2). MDCK control cells did not stain positive for surface IL-12 (Additional file 1: Figure S2A) or IL-23 (Additional file 1: Figure S2B). To prepare whole virus vaccines, MDCK stable transfectants were infected with influenza virus (A/PR/8/34) and virions bearing membrane-bound IL-12 (CYT-IVAC^~mIL12^) and IL-23 (CYT-IVAC^~mIL23^) were harvested from the supernatants, gradient-purified and subsequently inactivated using β-propiolactone (BPL) [15]. Non-adjuvanted whole inactivated virus (A/PR/8/34) WIV grown from influenza virus infected wild type MDCK cells was used as control in this study.

**Figure 1:**
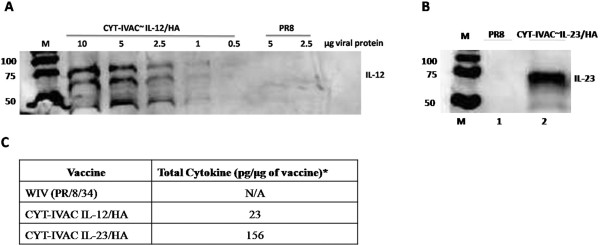
**Western blot analysis of CYT-IVAC**^**~IL12 **^**and CYT-IVAC**^**~IL23**^**.** Whole viral lysates were run on 12% SDS-PAGE gel, blotted on PVDF membrane and incubated with IL-12/23p40 antibody followed by anti-species secondary antibodies conjugated to HRP. **(A)** Dilutions of CYT-IVAC^~IL-12^ ranging from 10 μg to 0.5 μg of protein and **(B)** CYT-IVAC^~IL-23^ (lane 2) (5 μg) and PR8 (lane 1) (5 μg) were probed with anti-IL12/23 p40 antibody (eBioscience). **(C)** Quantitation of virus-incorporated cytokine (pg of cytokine per μg of vaccine) (FlowCytomix™, eBioscience).

Western blot analysis probed with antibodies specific for IL-12 or IL-23 was employed to validate full-length incorporation the heterodimeric cytokine fusion constructs into the specific CYT-IVAC formulations (Figure 1). Separation and staining of the CYT-IVAC^~mIL12^ and CYT-IVAC^~mIL23^ formulations respectively (Figure 1A,B) revealed a prominent band of approximately 70 kDa in molecular weight. The predicted molecular weights of membrane-bound IL-12 and IL-23 constructs are 68.93 and 66.87 kDa respectively. The cytokine specific bands were not detected in our control non-adjuvanted WIV formulation (PR8). HA incorporation was quantitated using western blot analysis [15] and quantitation of cytokines (IL-12 and IL-23) was performed (Figure 1C) using an IL12/IL23p40 specific bead assay as described by the manufacturer (eBioscience). Together, these data confirm that our CYT-IVAC formulations display full-length membrane-bound immunomodulators in direct context with full-length viral hemagglutinin and other virion-associated proteins.

To explore *in vivo* adjuvanticity, female Balb/c mice were immunized with BPL-inactivated control WIV (A/PR/8/34), CYT-IVAC^~mIL12^ and CYT-IVAC^~mIL23^ (n = 5/group) either intramuscularly (I.M.) or intranasally (I.N.). On day 21, all mice were administered a booster dose of vaccine (I.M.). The I.N. prime followed by the I.M. boost was employed to mimic priming of mucosal antibody responses elicited during infection, followed by subsequent stimulation of systemic immune responses that may only be marginally elicited by the mucosal route, yet are actively stimulated following parenteral vaccination. Based on total viral protein administered, animals received 165 ng/0.33 ng (I.M.) and 1 μg/165 ng (I.N.) of HA protein respectively during the prime/boost immunizations.

**Figure 2:**
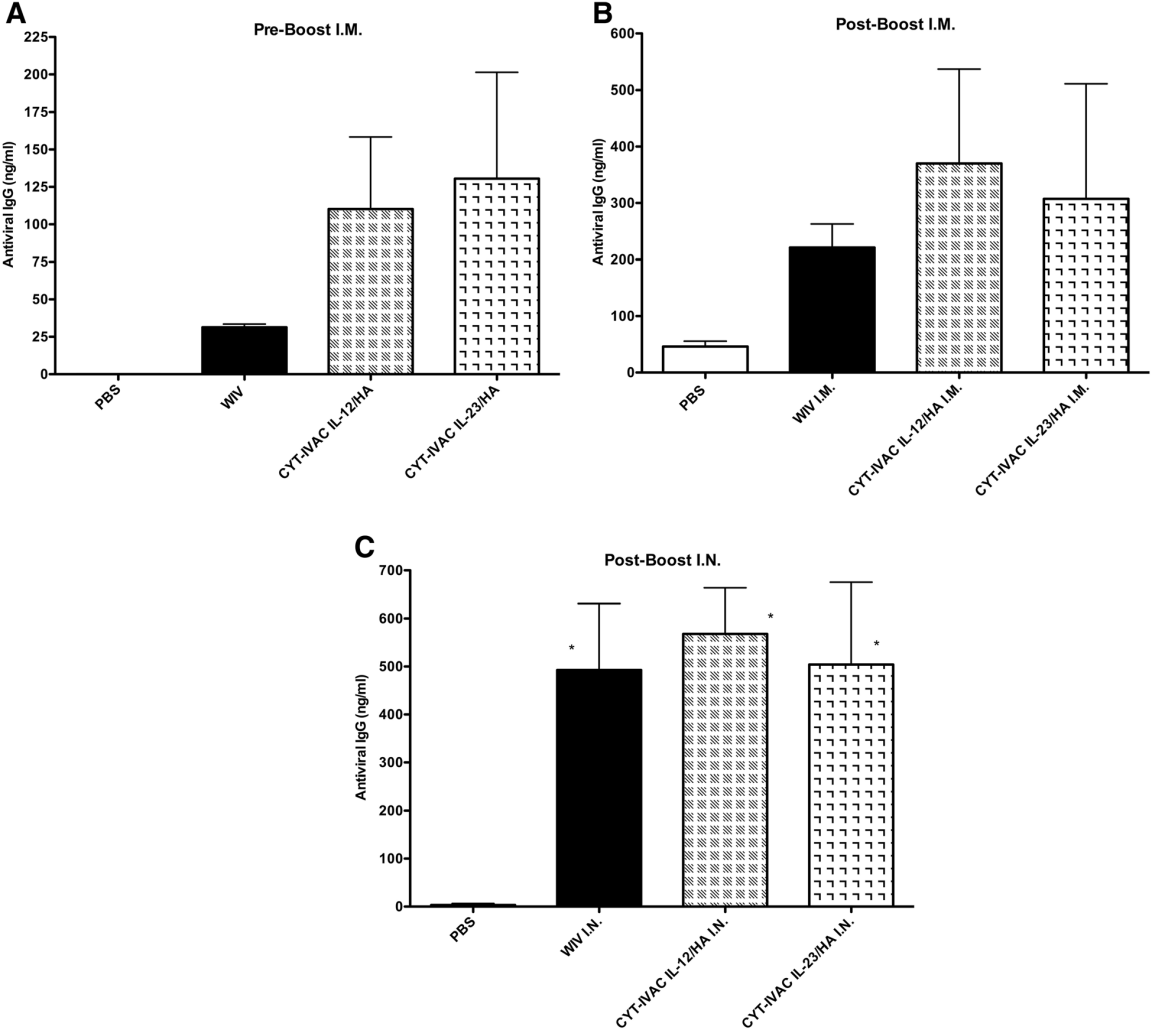
**Serum anti-viral IgG antibody responses.** 8–10 week old female Balb/c mice (Charles River Laboratories, CRL) were lightly anesthetized using isoflurane inhalant and immunized with β-propiolactone-inactivated double gradient purified control WIV (A/PR/8/34), CYT-IVAC^~IL-12^ and CYT-IVAC^~IL-23^ (n = 5) either intramuscularly (I.M.) in the right hindquarter or intranasally (I.N.) into the nostrils. PBS administered I.M. served as the sham-vaccinated group (n = 5). **(A)** Blood was collected pre-boost on day 19 **(B, C)**, post-boost on day 35 and serum antibody titers for influenza virus specific IgG were determined by ELISA. Data is displayed as the mean concentration ± SEM. (*p < 0.05 compared to PBS by One way ANOVA, Bonferroni’s multiple comparison test).

Anti-viral antibody levels elicited by CYT-IVACs and control non-adjuvanted WIV were determined on both pre-boost (day 19) and post-boost sera (day 35). As anticipated, I.M. immunization induced higher serum antiviral IgG responses as compared to the mucosal (I.N.) route supporting previous reports in both animal [24] and human [25] vaccine studies (Figure 2). Booster vaccination I.M. was given in all vaccine groups (both I.M. group and I.N. group) to boost primary responses and led to significantly higher antiviral IgG levels post-boost (40 ng/ml to 520 ng/ml) (Figure 2B) compared to pre-boost levels (25 to 205 ng/ml) within the I.M. group (Figure 2A). Interestingly, serum IgG antibodies were detected in the intranasal groups only following parenteral boosting (5 to 650 ng/ml). The levels were significantly greater compared to mock (PBS group) (*p < 0.05); albeit there were no significant differences among vaccinated groups (Figure 2C).

**Figure 3:**
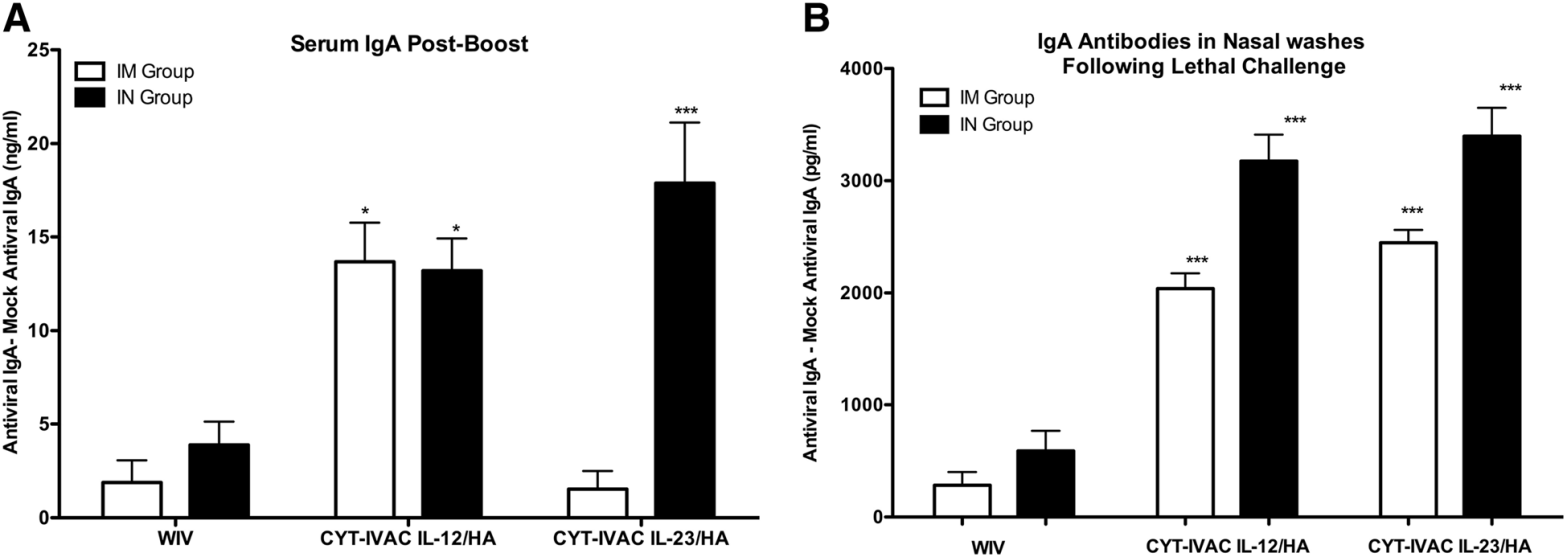
**CYT-IVAC**^**~IL12 **^**and CYT-IVAC**^**~IL23**^**boost mucosal anti-viral responses. (A)** Mice were vaccinated with either non-adjuvanted WIV or CYT-IVACs. Blood was collected post-boost on day 35 and serum antibody titers for influenza-specific IgA were determined by ELISA. **(B)** Mice were challenged with 100LD_50_ dose of mouse adapted A/PR/8/34 on day 36 following vaccination. At day 4 following challenge, animals (n = 5 mice/group) were sacrificed, nasal washes were collected in PBS (2 ml total volume) and flash frozen. Levels of influenza-specific IgA were quantitated by ELISA, with IgA standard curve extrapolation. Data represents mean concentration ± SE. **(A)** IM Group *p < 0.05 compared to WIV; IN Group *p < 0.05, ***p < 0.001 compared to WIV by One way ANOVA, **(B)** ***p < 0.001 compared to WIV, One way ANOVA Bonferroni’s multiple comparison test).

Further characterization of the antibody isotypes following booster vaccination revealed significantly higher levels of anti-viral serum IgA in animals that received CYT-IVAC^~mIL12^ and CYT-IVAC^~mIL23^ by the I.N. route (Figure 3A) compared to control WIV. Interestingly, I.M. administration of CYT-IVAC^~mIL12^ also induced significantly higher levels of serum anti-viral IgA. Nasal immunization of soluble cytokines IL-12, IL-1α, IL-18 with an HIV peptide has been shown to induce anti-HIV peptide serum and mucosal IgA [19]. This is the first report that membrane-bound IL-12 and IL-23 presented mucosally also stimulate serum anti-viral IgA.

All immunized animals were challenged on day 36 with 100 LD_50_ of mouse-adapted influenza virus A/PR/8/34 (H1N1) virus and sacrificed four days later for determining pre-existing vaccine induced protective responses. Since mucosal antibodies present in the respiratory tract are known to provide significant protection from influenza infection [26], we collected nasal washes from animals at day 4 post-infection and determined the anti-viral sIgA levels as a measurement of the vaccine-induced mucosal responses; anti-viral sIgA was not detectable in nasal washes of naïve challenged animals at day 4 (PBS group, Figure 3B). Both CYT-IVAC^~mIL23^ and CYT-IVAC^~mIL12^ vaccinated groups exhibited significantly higher levels of influenza-specific sIgA (p < 0.001) compared to the control WIV group, suggesting that both serve as potent mucosal adjuvants when co-presented in a membrane-bound formulation in direct context with WIV.

Surprisingly, high influenza-specific sIgA levels were also detected in the nasal washes of animals that received CYT-IVAC^~mIL23^ and CYT-IVAC^~mIL12^ strictly by the I.M. route (Figure 3B). This is not without precedence, as the transcutaneous or epicutaneous route of immunization coupled with mucosal adjuvants including bacterial toxins, chemical enhancers of cyclic AMP and the active form of Vitamin D3 have been reported to induce peripheral DC migration to Peyers patches (mucosal tissues) and subsequent priming of T and B cells for mucosal homing [27-29]. Hence, our membrane-anchored IL-12 and IL-23 adjuvants may be activating dendritic cells in muscle tissue and targeting them to migration in draining lymph nodes, in a state that is conducive for priming T and B cells for homing to mucosal tissues.

**Figure 4:**
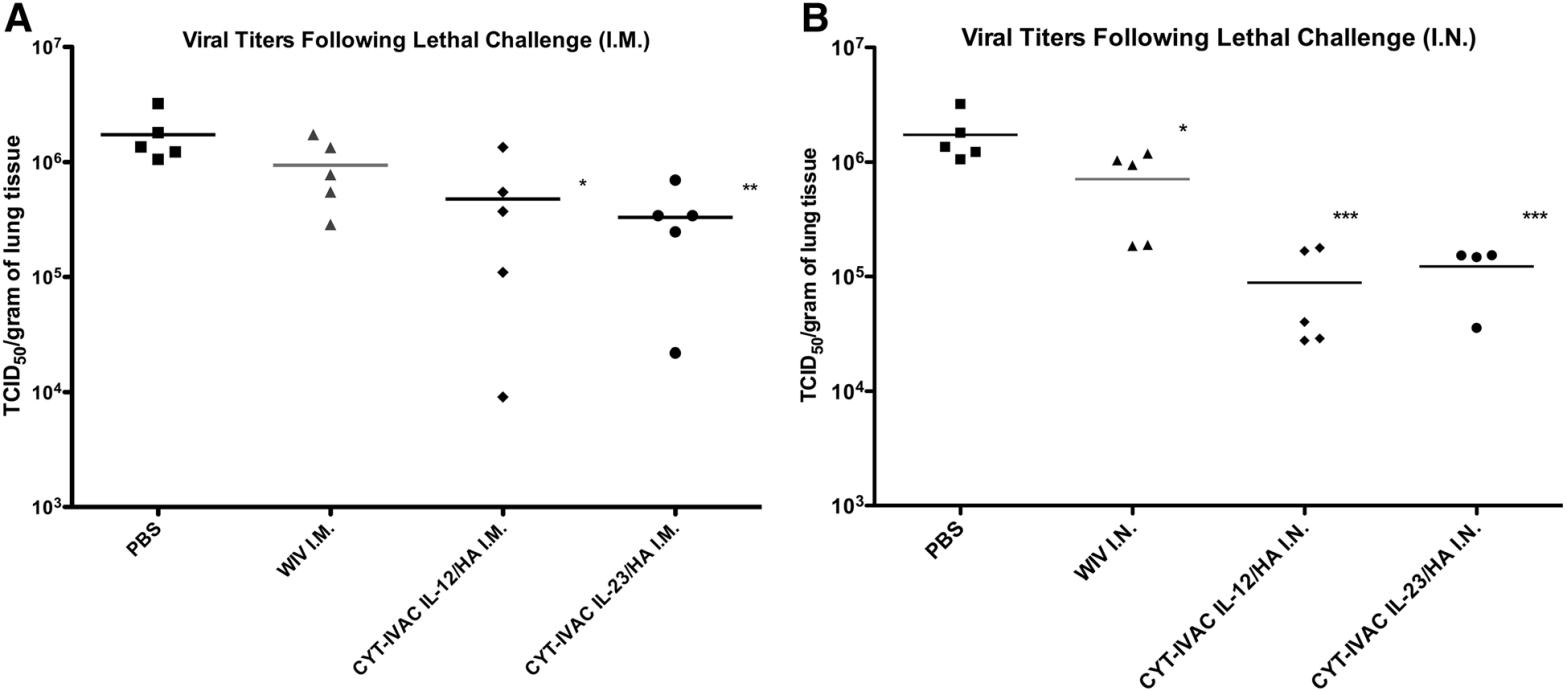
**CYT-IVAC immunization reduces viral lung burden following challenge.** Animals vaccinated I.M. **(A)** or I.N. **(B)** were challenged with a 100LD_50_ dose of mouse-adapted A/PR/8/34 on day 36 following vaccination. At day 4 following challenge, animals (n = 5) were sacrificed, lung tissues were collected and flash frozen. Viral loads from homogenized lung tissue (n = 5) were determined by tissue culture infectious dose assay. Data is expressed as TCID_50_ per gram of lung tissue. (*p < 0.05, **p < 0.01, ***p < 0.001 compared to PBS, one way ANOVA, Bonferroni’s multiple comparison test.

A reduction in viral titers in lung tissue of vaccinated animals following viral challenge serves as a valuable correlate of protective efficacy of viral vaccines [30]. Whereas I.M. vaccination of either CYT-IVAC^~mIL23^ or CYT-IVAC^~mIL12^ was able to reduce viral lung loads at day 4 post-challenge compared to non-vaccinated controls, they only trended towards a better protective response compared to non-adjuvanted WIV (Figure 4A). Interestingly, we found that recall IgA levels in nasal washes were significantly higher in both CYT-IVAC groups than the WIV non-adjuvanted group following lethal challenge irrespective of the route of administration. These responses may be responsible for some of the variation of viral lung loads observed in animals receiving CYT-IVACs by the IM route. However, this also suggests that there are fundamental differences in the specificity of mucosal induced anti-viral IgA compared to anti-viral IgA responses induced at other parenteral sites.

Mucosal administration of CYT-IVAC^~mIL23^ or CYT-IVAC^~mIL12^ led to significantly reduced viral loads upon challenge compared to the non-adjuvanted WIV control group (Figure 4B); these data correlated well with the observed high levels of sIgA detected in serum and the nasal wash of these animals. This is the first time that the membrane-bound IL-12 or IL-23 co-presented directly on influenza WIV has been found to induce robust mucosal immune responses. Soluble IL-12 as a nasal adjuvant is well documented [6] and has been reported to stimulate mucosal sIgA and systemic IgG antibody as well as cellular responses in a variety of vaccine formulations [8]. Our data supports these observations, but also suggest that even low concentrations of IL-12 (picograms) can be used in a membrane-bound formulation to boost mucosal sIgA levels. IL-23 inducing mucosal antibody responses is a novel observation and clearly further work is required to address the mechanism. Based on previous reports of IL-17 influencing B cell activation [31] and germinal center development [32], the IL-23 bearing CYT-IVAC could be modulating Th17 responses. Recently, activation of lung Th17 cells were also found to induce the development of polymeric Ig receptor and elevate mucosal secretion of IgA antibodies [33]. Besides IgA antibodies, anti-viral IgG in BAL fluids as well as T cell responses all contribute towards protection and recovery from viral challenge.

Our approach of using cytokines in direct context with WIV vaccines involves very low amounts of cytokine (picograms per microgram of vaccine). This approach may potentially lower the amount of antigen required to induce protection and may also lower the incidence of adverse side effects reported for WIVs. In most studies soluble cytokines have been administered in μg amounts, which is clearly well in excess of the amounts utilized in the present study; albeit the total number of doses and dosing schedules vary significantly depending on the study (reviewed extensively in [8,10]. It should be noted that soluble IL-23 has been reported to have adverse effects such as inducing chronic inflammation and autoimmune disease [34,35] and both antitumor and tumor promoting effects [36,37]. Our approach that limits systemic distribution of cytokines may overcome some of the safety issues and potential autoimmune inflammatory reactions from soluble IL-23.

In summary, this is the first report of the nasal adjuvanticity of both IL-12 and IL-23 in a membrane-bound formulation co-presented with WIV. In light of our recent study using our CYT-IVAC approach in “aged” mice, we hypothesize that both the IL-12 and IL-23 expressing CYT-IVAC formulations may show even more promise as a mucosal formulation for the elderly, but this will require further more in depth investigation. Finally, this study revisits the use of WIV vaccine formulations adjuvanted for mucosal delivery. Future experiments focusing on evaluating protective efficacy of dose sparing with mucosally administered CYT-IVAC^~IL-12^ and CYT-IVAC^~IL-23^ vaccines in both young and old preclinical challenge models are needed to provide greater insights on the efficacy of these vaccines.

## Abbreviations

CYT-IVAC: Cytokine bearing influenza vaccine; WIV: Whole inactivated virus influenza vaccine; I.M.: Intramuscular; I.N.: Intranasal; IL-12: Interleukin-12; IL-23: Interleukin-23.

## Competing interests

The authors declare that they have no competing interests.

## Authors’ contributions

TK was responsible for construct design and assembly, establishing MDCK producer cell lines, vaccine production and characterization, completion of serological assays (ELISA), design and completion of vaccine efficacy studies, overall study design, analysis and interpretation of results, statistical analysis, drafting and reviewing the manuscript. LH participated in animal experimental design and execution. KPH participated in study design and interpretation. PCR conceived the study, served as the principle investigator, participated in study design, aided in interpretation of results, helped to draft and review the manuscript. All authors read and approved the final manuscript.

## Supplementary Material

Additional file 1: Figure S1 Pictorial diagram of CYT-IVAC gene constructs. (A) CYT-IVAC^IL-12^fusion gene construct (1620 bp) was composed of mIL-12p35p40 subunits joined by short hydrophobic linker molecule(Li) fused in frame to the a short 222 bp coding region of the Hemagglutinin (HA) gene derived from Influenza virus (A/WSN/33), which encodes for a short extracellular stalk region and the transmembrane and cytoplasmic tail region of the HA (HA1513). (B) The CYT-IVAC^IL-23^ fusion gene construct was constructed in a similar fashion. Both cytokine genes were subcloned into the pcDNA3.1 expression vector, placed under control of the CMV promoter (pCMV). **Figure S2.** Indirect immunofluorescence cell surface staining of MDCK transfectant cell lines cell lines constitutively expressing mIL-12/HA (C) and mIL-23/HA (D) with MDCK cells serving as negative control (A,B) using cytokine specific antibodies. Click here for file
