# Supplementary material for: Membrane-bound IL-12 and IL-23 serve as potent mucosal adjuvants when co-presented on whole inactivated influenza vaccines
Source: Virol J. 2014 May 3;11:78. doi: 10.1186/1743-422X-11-78 (PMC4036309; doi:10.1186/1743-422X-11-78)
Supplement: Additional file 1: Figure S1 — Pictorial diagram of CYT-IVAC gene constructs. (A) CYT-IVACIL-12fusion gene construct (1620 bp) was composed of mIL-12p35p40 subunits joined by short hydrophobic linker molecule(Li) fused in frame to the a short 222 bp coding region of the Hemagglutinin (HA) gene derived from Influenza virus (A/WSN/33), which encodes for a short extracellular stalk region and the transmembrane and cytoplasmic tail region of the HA (HA1513). (B) The CYT-IVACIL-23 fusion gene construct was constructed in a similar fashion. Both cytokine genes were subcloned into the pcDNA3.1 expression vector, placed under control of the CMV promoter (pCMV). Figure S2. Indirect immunofluorescence cell surface staining of MDCK transfectant cell lines cell lines constitutively expressing mIL-12/HA (C) and mIL-23/HA (D) with MDCK cells serving as negative control (A,B) using cytokine specific antibodies. [file 1743-422X-11-78-S1.docx]

**
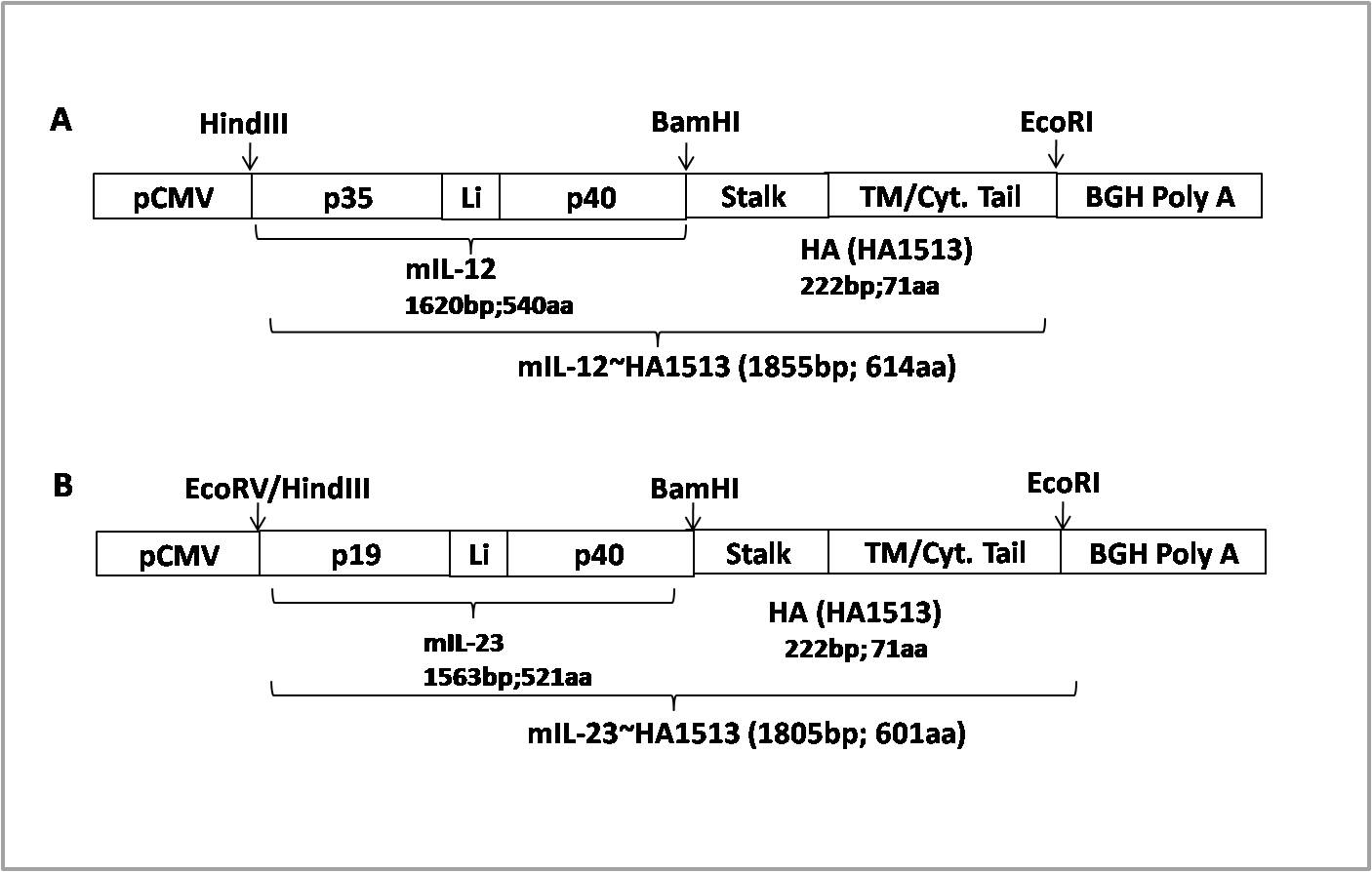
**

**Supplemental S1.** Pictorial diagram of CYT-IVAC gene constructs. (A) CYT-IVAC^IL-12^ fusion gene construct (1620 bp) was composed of mIL-12p35p40 subunits joined by short hydrophobic linker molecule (Li) fused in frame to the a short 222 bp coding region of the Hemagglutinin (HA) gene derived from Influenza virus (A/WSN/33), which encodes for a short extracellular stalk region and the transmembrane and cytoplasmic tail region of the HA (HA1513). (B) The CYT-IVAC^IL-23^ fusion gene construct was constructed in a similar fashion. Both cytokine genes were subcloned into the pcDNA3.1 expression vector, placed under control of the CMV promoter (pCMV).

**
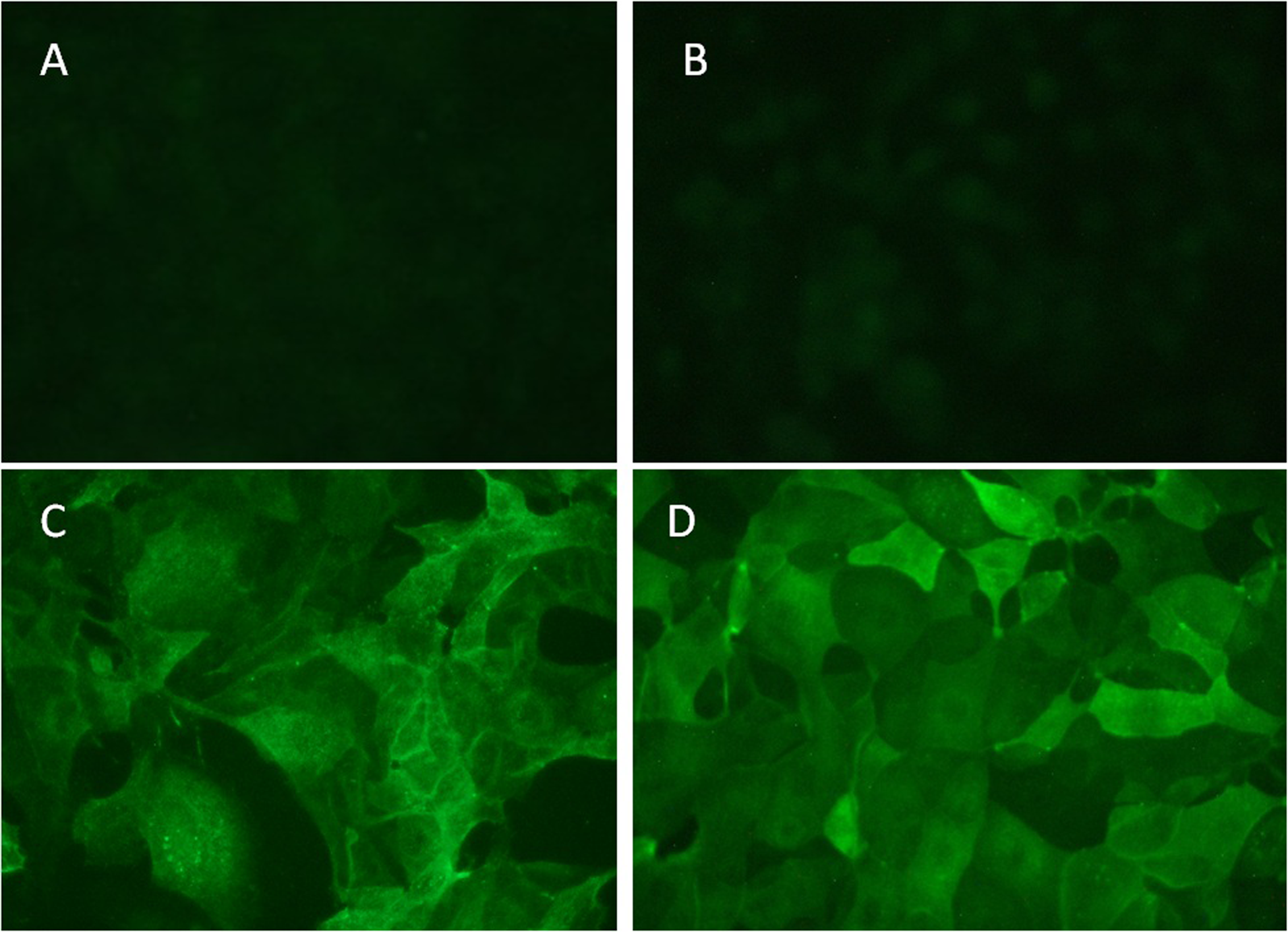
**

**Supplemental S2.** Indirect immunofluorescence cell surface staining of MDCK transfectant cell lines cell lines constitutively expressing mIL-12/HA (C) and mIL-23/HA (D) with MDCK cells serving as negative control (A, B) using cytokine specific antibodies.
